# The Effects of Blast Exposure on Protein Deimination in the Brain

**DOI:** 10.1155/2017/8398072

**Published:** 2017-05-24

**Authors:** Peter J. Attilio, Michael Flora, Alaa Kamnaksh, Donald J. Bradshaw, Denes Agoston, Gregory P. Mueller

**Affiliations:** ^1^Neuroscience Program, Uniformed Services University of the Health Sciences, Bethesda, MD, USA; ^2^Department of Anatomy, Physiology and Genetics, Uniformed Services University of the Health Sciences, Bethesda, MD, USA

## Abstract

Oxidative stress and calcium excitotoxicity are hallmarks of traumatic brain injury (TBI). While these early disruptions may be corrected over a relatively short period of time, long-lasting consequences of TBI including impaired cognition and mood imbalances can persist for years, even in the absence of any evidence of overt injury based on neuroimaging. This investigation examined the possibility that disordered protein deimination occurs as a result of TBI and may thus contribute to the long-term pathologies of TBI. Protein deimination is a calcium-activated, posttranslational modification implicated in the autoimmune diseases rheumatoid arthritis and multiple sclerosis, where aberrant deimination creates antigenic epitopes that elicit an autoimmune attack. The present study utilized proteomic analyses to show that blast TBI alters the deimination status of proteins in the porcine cerebral cortex. The affected proteins represent a small subset of the entire brain proteome and include glial fibrillary acidic protein and vimentin, proteins reported to be involved in autoimmune-based pathologies. The data also indicate that blast injury is associated with an increase in immunoglobulins in the brain, possibly representing autoantibodies directed against novel protein epitopes. These findings indicate that aberrant protein deimination is a biomarker for blast TBI and may therefore underlie chronic neuropathologies of head injury.

## 1. Introduction

Central features in traumatic brain injury (TBI) include oxidative stress [1–4], breakdown of the blood brain barrier [5, 6], and a protracted period of Ca^2+^ excitotoxicity [7, 8]. These early consequences of brain injury set the stage for the progressive development of long-term pathologies including impaired learning and memory, as well as emotional and mood imbalances [9–13]. These long-term consequences of TBI can be complex and may increase in severity over months and years, even though the injury may have been classified as clinically mild, and there is no evidence of physical injury using the most sensitive of imaging techniques [14, 15]. At present, there is a gap in our knowledge linking the acute events of mild TBI to chronic pathology. Importantly, repeated mild TBI has now been identified as the most significant environmental factor for developing chronic neuropsychiatric symptoms [16–18].

**Figure 1:**
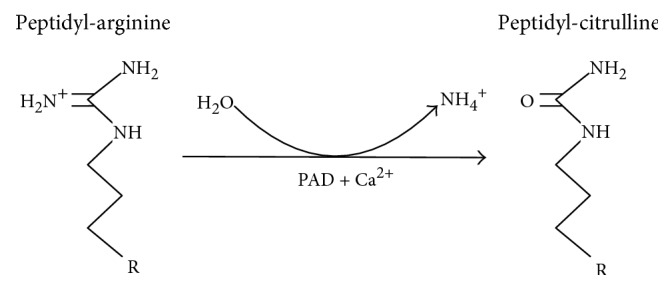
Protein deimination is catalyzed by a family of structurally related, calcium-dependent enzymes known as peptidylarginine deiminases (PADs). Protein deimination involves the conversion of an intraprotein arginine residue to a citrulline residue, resulting in the loss of a positively charged amine group and 1 Da in molecular mass.

The purpose of this study was to determine if aberrant deimination of brain proteins occurs in response to TBI and, therefore, potentially contributes to the long-term consequences of TBI. Deimination, or citrullination, is a posttranslational modification involving the calcium-dependent conversion of peptidyl-arginine to peptidyl-citrulline catalyzed by peptidylarginine deiminase (PAD) (Figure 1). This modification can result in the creation of novel, potentially antigenic epitopes that can elicit autoimmune responses [19, 20] (Figure 1). Specifically, disordered deimination of the joint proteins, filaggrin [21] and vimentin [22], generates antigenic epitopes [23] which can trigger a sustained autoimmune attack that eventually destroys the synovial compartment [24]. Disorders in protein deimination are also implicated in the diseases of the central nervous system, most notably multiple sclerosis [25–27], where the deimination of myelin basic protein appears to underlie a sustained autoimmune attack against the deiminated protein [28]. There is increasing interest in the possibility that the immune system plays a role in the long-term pathogenesis of TBI [29, 30].

It was previously reported that controlled cortical impact in rodents selectively alters the deimination status of a subset of proteins constituting the brain proteome [31], presumably due to injury-induced conditions of oxidative stress and calcium excitotoxicity. The present investigation was designed to extend these findings to a large animal model using blast injury as a noninvasive form of TBI. As seen with direct cortical injury in rodents [31], only a small subset of the entire brain proteome underwent blast-induced deimination in the porcine brain. Two of the six proteins identified as being deiminated were vimentin and glial fibrillary acidic protein (GFAP). The deimination sites found within vimentin and GFAP corresponded to previously reported sites of deimination, respectively, in rheumatoid arthritis and in multiple sclerosis [32] and Alzheimer's disease [33, 34]. Moreover, the levels of immunoglobulin G (IgG) detected in the brains of blast-exposed animals were markedly elevated as compared to those present in control animals, possibly representing autoantibodies directed against novel protein epitopes. These findings indicate that aberrant protein deimination may be a biomarker for blast TBI and may therefore underlie chronic neuropathologies through mechanisms involving the adaptive immune system.

## 2. Materials and Methods

### 2.1. Animals

Studies were conducted in adult male Yucatan miniature and Yorkshire swines (Sinclair BioResources, LLC., and Archer Farms, Darlington, MD, respectively) weighing 40–50 kg, *N* = 4/group). Animals were cared for and treated in accordance with guidelines approved by the US Department of Agriculture and the Medical Research and Material Command of the US Army.

Anesthetized pigs in the injured group were positioned in sternal recumbency and equipped with a specially made Kevlar and lead body armor as well as head and face protection. Animals were then transferred to a blast tube, simulating free-field blast, where they received a single moderate blast overpressure exposure (40–52 psi, average = 46 psi). Details pertaining to anesthesia, pre- and postprocedural treatments, and blast structure were as described earlier [5, 35–37]. Immediately after blast exposure, animals were removed from the blast structure and returned to the adjacent procedure facility for recovery. The endotracheal tube was maintained until animals exhibited normal pharyngeal function via cough reflex. Physiological parameters were electronically recorded by the monitoring system until animals were fully recovered from anesthesia and returned to their holding cages. In the days that followed, animals were assessed for pain or distress and monitored for general health as well as cranial nerve and neurologic and respiratory functions.

All animals were euthanized 2 weeks after exposure, and whole brains were collected and rinsed in physiologic saline. Coronal sections were prepared (0.75 inches thick), snap frozen on dry ice, and stored at −80°C until used.

### 2.2. Sample Preparation

Brain sections of frontal cortex were thawed and further dissected to produce wedges of tissue that contained an equivalent representation of all layers of the cerebral cortex frontal lobe. Tissue samples were homogenized in 5 volumes/tissue weight of 0.1 M Tris buffer (pH 7.4) containing 1x complete protease inhibitors using a Polytron (setting 6; 3 × 15 second pulses, with chilling in between) (Roche, Basel, Switzerland) followed by 3 freeze-thaw cycles and centrifugation (20000 ×g, 15 min, 4°C). The resulting supernatants were removed and stored at −80°C until used.

### 2.3. Liquid-Phase Isoelectric Focusing

Liquid-phase isoelectric focusing (LP-IEF) of brain supernatants was carried out as previously described [31]. Briefly, treatment group pools (naïve and blast, *N* = 4/group) underwent concentration and buffer exchange to water/1x protease inhibitors by Vivaspin (10 kDa molecular weight cut off (MWCO); General Electric, Fairfield, CT), removing TRIS which interferes with LP-IEF. Samples were then diluted in 1.1x IEF running solution (7.7 M urea, 2.2 M thiourea, and 4.4% CHAPS) and 1x complete protease inhibitor (1 part sample/9 parts IEF buffer). Samples were further adjusted for IEF fractionation by combining 900 *μ*L pooled sample with ampholytes (150 *μ*L, pH 3–10; Novex, Thermo Fisher, ZM0021; Waltham, MA), dithiolthreitol (DTT; 25 *μ*L, 4 M), and bromphenol blue (20 *μ*L, 10 mg/mL). IEF fractionation was performed under the following conditions: (1) 100 V, 2 mA, 2 W (20 min); (2) 200 V, 2 mA, 2 W (80 min), (3) 400 V, 2 mA, 2 W (80 min), and (4) 600 V, 2 mA, 2 W (80 min) using the ZOOM IEF Fractionator (Thermo Fisher, Waltham, MA). The resulting fractionation produced samples corresponding to the predicted IEF pH ranges for the fractionator (pH 3.0–4.6, pH 4.6–5.4, pH 5.4–6.2, pH 6.2–7.0, and pH 7.0–9.1) as judged by pH testing using pH strips. 1-dimensional gel electrophoresis and Coomassie staining (see below) were used to confirm equivalent fraction profiles for the naïve and blast samples and to verify equivalent protein concentrations for the naïve and blast samples of the same pH range.

### 2.4. 1-Dimensional Gel Electrophoresis

IEF fractions were further resolved by molecular weight fractionation using conventional sodium dodecyl sulfate polyacrylamide gel electrophoresis (SDS-PAGE). This two-step reduction in the complexity of the proteome by IEF and SDS-PAGE was important for the visualization of deiminated proteins by western blot analysis. Briefly, IEF samples were diluted in 4x reducing loading buffer (10% LDS, 10% glycerol, 0.4 M DTT, 250 mM Tris buffer, 20 *μ*L bromphenol blue (10 mg/mL), pH 8.4), heated at 70°C (10 min), and then fractionated in NuPage 4–12% Bis-Tris gels (Novex, Thermo Fisher, Waltham, MA), using 1x MES (2-[N-morpholino] ethanesulfonic acid) running buffer (9.76 gm/L MES, 60.6 gm/L Tris Base, 0.3 gm/L disodium ethylenediaminetetraacetic acid (EDTA), and 1 gm/L SDS, pH 8). Proteins were transferred to nitrocellulose using an iBlot transfer system (Thermo Fisher, Waltham, MA).

### 2.5. Immunoblotting

#### 2.5.1. Protein Deimination

Nitrocellulose membranes were blocked with 5% nonfat dry milk/Tris-buffered saline/Tween 20 (TBS-T) (25 mM Tris Base, 0.115 M NaCl, 25 mM KCl, 0.1% Tween20, and pH 7.5) for 2 h at room temperature and then incubated overnight at 4°C with mouse monoclonal anti-protein citrulline primary antibody 6B3 [31, 38] (stock = 1.79 mg/mL) diluted (1 : 500) in 5% nonfat dry milk/TBS-T. Membranes were then washed in TBS-T (3 times over 60 min), incubated with secondary antibody (HRP-conjugated goat anti-mouse IgG (H+L), 31430, 1 : 2500 in TBS-T; Thermo Fisher, Waltham, MA) at room temperature for 2 h. Membranes were then washed in TBS-T (3 times over 60 min) and then visualized with enhanced chemiluminscence (ECL) (Novex ECL HRP Chemiluminescent Substrate Reagent Kit; WP20005, Invitrogen, Thermo Fisher, Waltham, MA) using the ChemiDoc Touch imaging system (Bio-Rad Laboratories, Hercules, CA). The specificity of the 6B3 mAb for detecting deiminated proteins was verified as described previously [31]. Images collected were analyzed using Image Lab software (v5.2.1, Bio-Rad Laboratories; Hercules, CA). Anti-peptidyl-citrulline, clone F95 antibody, was obtained commercially from Millipore (ab# MABN328, Darmstadt, Germany) and used similarly.

#### 2.5.2. Tissue IgG

Nitrocellulose membranes were blocked with 5% nonfat dry milk in TBS-T (2 h, room temperature) and then incubated with goat anti-porcine IgG (H+L), HRP-conjugated antibody (1 : 2500 in TBS-T, EMD Millipore, Billerica, MA, AP166P) at room temperature for 2 h. Membranes were then washed in TBS-T (3 times over 60 min) and then visualized and analyzed as described for protein deimination above. Quantitation of the ECL Western blot signals was based upon standardization to protein load for each sample, as determined by the signal density for equivalent samples visualized on Coomassie-stained gels. ImageJ (Mac Version 1.50i, National Institutes of Health) was used to determine both ECL and Coomassie data; signal densities of immunoreactive ECL features (heavy and light chain bands) were summed and adjusted for protein load based upon a percent difference from a reference protein load (highest protein load of control samples = 100%).

### 2.6. Protein Identification and Mapping of Deimination Sites

Immunoreactive signals of interest were mapped to corresponding banding patterns of the Coomassie-stained gels. The bands were excised and analyzed by peptide mass finger printing and tandem mass spectrometry (MS-MS) using the proteomic services of the W.M. Keck Foundation Biotechnology Resource Laboratory (New Haven CT, USA). Briefly, gel bands were cut into smaller pieces, digested with trypsin, peptides extracted and desalted, and analyzed by liquid chromatography (LC) MS-MS using an Orbitrap Fusion Tribrid mass spectrometer (Thermo Fisher, Waltham, MA). Both MS and MS/MS scans were acquired in an Orbitrap analyzer. MS scans were of m/z range 350–1550, resolution of 120000, AGC (automatic gain control) target of 2e5, and maximum injection time of 60 ms, while MS/MS scans were with fixed first mass of m/z 120, resolution of 60000, AGC target of 5e4, and maximum injection time of 110 ms. Precursor ions were fragmented by high energy collision-induced dissociation collision energy (%) set to 28.

The raw files were processed using Proteome Discoverer v2.1 software (Thermo Fisher, Waltham, MA). The files were searched with Sequest HT algorithm against pig UniProt database (downloaded June 2016). The fragment ion mass tolerance of 0.02 Da, parent ion tolerance of 10 ppm, and digestion enzyme trypsin were specified in the Sequest analysis parameters. Oxidation of methionine, deamidation of asparagine and glutamine, deimination (citrullination) of arginine, and propionamide of cysteine were specified in Sequest as variable modifications.

Scaffold software (v4.6.1, Proteome Software) was used to validate peptide and protein identifications. Initial protein analysis was performed with a protein threshold of 99%, a minimum of 3 peptides, and a peptide threshold of 95%. The inclusion criteria for peptides with a deiminated arginine were an Xcorr of 2 or more and a deltaCn of >0.4. Peptides meeting these criteria were then assessed for a 43 Da neutral loss assessing for the loss of isocyanic acid (HNCO) through spectrometric analysis described by Hao et al. [37]. All spectra were visually reviewed to insure quality and clear presence of the 43 Da neutral loss signature for deimination.

The expected mass of the neutral loss of isocyanic acid was determined by subtracting the product of a 43 Da loss divided by the peptide charge from the observed mass of the peptide sequence [37]. Peaks corresponding to the expected neutral loss were identified in the spectrum data and included if an observed peak was less than 2 Da from the expected peak. The only exception to this was GABA transaminase (4-aminobutyrate aminotransferase) that had peaks identified at approximately 3 Da from the expected neutral loss mass. This tentative identification was included here based on the high quality of the spectrum, Xcorr, and deltaCn.

### 2.7. Statistical Analysis

The quantitation of the IgG ECL western blot signals was based on standardization to protein load for each sample, as determined by the signal density for equivalent samples visualized on Coomassie-stained gels. ImageJ (Mac version 1.50i, National Institutes of Health) was used to determine both ECL and Coomassie data; signal densities of immunoreactive ECL features (heavy and light chain bands) were summed and adjusted for protein load based on a percent difference from a reference protein load (highest protein load of control samples = 100%). Analysis of the combined relative signal intensity of the IgG naïve and blast results was performed using Prism 7 for Mac OS X (v7.0a, GraphPad Software Inc.). An unpaired *t*-test was performed with the standard variance assumed to be equal in the population. Statistical significance was determined using the Holm-Sidak method with an alpha of 0.05.

## 3. Results

**Figure 2:**
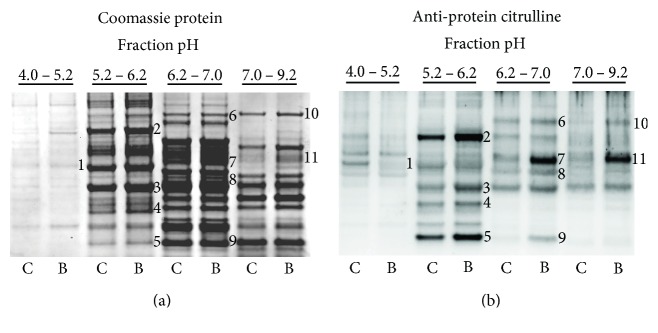
Blast-induced deimination of proteins in porcine brain. Brain samples were collected 2 weeks following a single blast exposure (average pressure = 46 psi). Homogenates of control (C) and the blast-exposed (B) cerebral cortex were prefractionated by LP-IEF. The resulting pH fractions were further fractionated by 1-dimensional SDS-PAGE (a) and analyzed for protein deimination by western blotting (b) using the mouse monoclonal 6B3 anti-protein citrulline antibody. Immunoreactive features affected by TBI (numbered, panel (b)) were mapped to corresponding bands in a Coomassie-stained protein gel (numbered, panel (a)). These were collected, identified, and mapped for site-specific deimination by peptide mass fingerprinting using liquid chromatography and tandem mass spectrometry (LC MS/MS).

Figure 2 shows the effects of blast exposure on the status of protein deimination in the porcine cerebral cortex. Brains were collected 2 weeks postblast exposure, and cerebral cortex homogenates were prepared from each subject. Treatment group pools of sham and blast samples, representing 4 animals each, underwent two-dimensional fractionation involving liquid phase isoelectric focusing (LP-IEF) followed by molecular weight fractionation using 1-dimensional sodium dodecyl sulfate polyacrylamide gel electrophoresis (SDS-PAGE). These steps for reducing the complexity of the proteome were necessary to clearly reveal western blot signals in the analyses of protein deimination. Panel (a) shows the Coomassie-stained protein profiles for the control and blast groups over the four LP-IEF pH fractions. The data show that LP-IEF yielded pH fractions with distinct protein profiles, indicating the effectiveness of the IEF procedure to separate a complex proteome into subfractions having reduced protein complexity. It was also observed that within a given pH fraction, there was no apparent difference in the Coomassie-banding profile between the control and blast samples with the exception of feature 11. This feature, which had increased Coomassie staining in the blast-exposed fraction, was determined to contain IgG by both mass spectrometric and western blot analyses.

The effects of blast exposure on the profile of protein deimination, using 6B3 antibody western blotting, is shown in panel (b). These data indicate that there is a basal level of protein deimination in the control condition that involves a small subset of the proteins making up the entire brain proteome. Further, blast exposure dramatically affected the deimination status of several, but not all of these features. In most cases, blast exposure resulted in a pronounced increase in the observed deimination signal (features 2–11), in some cases increasing from virtually no signal in the control condition (feature 9). There was also evidence for blast exposure in reducing the degree of protein deimination within a protein band, as can be most clearly seen for feature 1. Preliminary findings with an alternative anti-protein citrulline antibody, F95, identified some features that were not revealed by antibody 6B3 and vice versa (not shown), suggesting that the amino acid context of a deimination site contributes to its antibody recognition. Additionally, it was observed that deimination signals observed by western blot were reduced upon repeated freeze-thaw cycles of the samples, indicating the importance of preparing sample aliquots for storage and repeat analyses.

**Figure 3:**
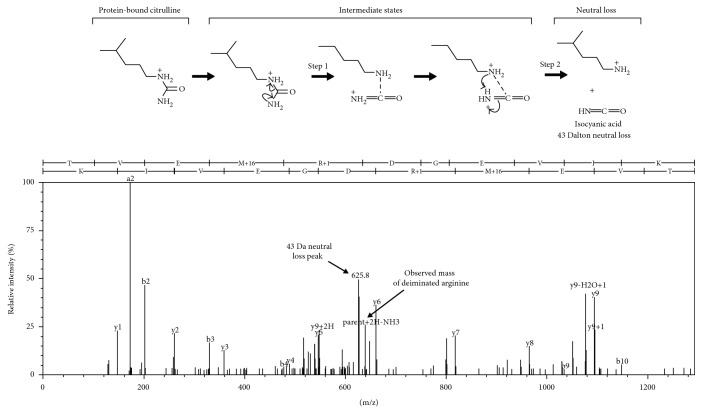
Mapping of protein deimination sites by neutral loss. Tryptic peptides were fragmented by collision-induced dissociation, and resulting spectra were analyzed for a neutral loss of 43 Da, reflecting the loss of isocyanic acid as a fragmentation product of citrulline (upper panel). The representative spectrum shown here depicts the Y and B ion spectra of GFAP peptide, TVEMrDGEVIK, with the neutral loss peak observed for the deiminated arginine (r) at 625.8 Da. Because the parent peptide ion was doubly charged in this case, the observed neutral loss in the spectrum was 21.5 Da, reflecting 43 Da/2.

**Figure 4:**
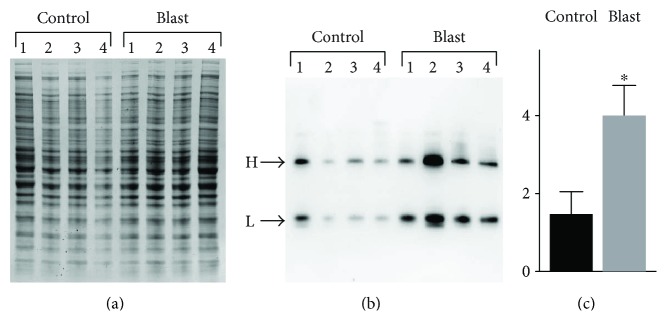
Effects of blast exposure on the presence of IgG expression in the cerebral cortex of swine. Homogenates of control and the blast-exposed (2 weeks postinjury) cerebral cortex (N-4/group) were fractionated by (a) 1-dimensional SDS-PAGE and (b) analyzed for IgG content by western blotting. Immunoreactive heavy (H) and light (L) chain IgGs were visualized using an anti-porcine IgG detection antibody. The values for the total IgG chemiluminescence signal (H+L) (c) for each sample were standardized to protein load (a) by densitometry analysis using ImageJ, and resulting values were analyzed statistically. The relative signal intensity is shown on the *y*-axis as densitometry values ×100. Data are presented as the mean ± standard error; ^∗^*p* ≤ 0.005.

Immunoreactive features identified by 6B3 western blotting (panel (b), features 1–11) were mapped to corresponding protein bands in a replicate Coomassie-stained protein gel (panel (a), features 1–11). These bands were collected and analyzed proteomically to identify the proteins present and to map their respective deimination sites. Site-specific deimination was confirmed by the demonstration of neutral loss of 43 Da representing the signature deimination fragment isocyanic acid [37] (Figure 3) (see Materials and Methods for details).  Table 1 presents a list of the 6 proteins definitively identified and their respective deimination sites. The findings include deiminated GFAP and vimentin, both of which are recognized as autoantigens in neuropathology [39] and rheumatoid arthritis [40], respectively. Western blotting for protein deimination employed an anti-mouse IgG detection antibody that was subsequently shown to cross-react with porcine IgG. This reagent revealed an intense signal in feature 11 of the blast brain pool (Figure 2 feature 11) that was not pronounced in the control pool. Proteomic analysis determined that the dominant protein in this immunoreactive feature was, indeed, porcine IgG as opposed to another protein that had reacted with the 6B3 primary antibody. The increased presence of IgG in the injured cortex was further verified by using a separate detection antibody specific to porcine IgG (Figure 4), suggesting that an adaptive immune response to blast injury may have occurred in these animals. An analysis of the individual samples making up the pools of naïve and blast-injured brain tissue further confirmed that blast injury was associated with significantly elevated levels of IgG in the cerebral cortex. The Coomassie-stained protein profile for each sample is presented in panel (a). The corresponding western blot for porcine IgG is shown in panel (b) (Figure 4). Panel (c) represents an integration analysis of the western blot signal intensities for IgG heavy and light chains (panel (b)), standardized to total protein load (panel (a)). The data show that blast significantly increased the amount of IgG detected in the cerebral cortex of blast-exposed swine. Variations in this response were observed among subjects, possibly reflecting variations in the degree of injury caused by the blast exposure.

## 4. Discussion

Brain injury can result in long-term symptomologies that include impaired learning and memory; poor concentration/attention; slowed thinking; emotional and mood imbalances including increased anxiety, depression, disorientation, and headaches; and emotional and cognitive dysfunction. These problems can persist for years after injury, often in the absence of any detectable neuropathology [14, 15]. At the cellular level, however, brain injury can result in a sustained state of neuroinflammation that is reflected in the proinflammatory, M1 phenotype of microglia [41, 42]. The persistence of these responses is consistent with the involvement of the adaptive immune system [29, 30]. The present findings raise the possibility that aberrant deimination of specific brain proteins, with the resulting generation of antigenic epitopes, may be an important mechanism in this phenomenon.

The data presented here demonstrate that blast injury upregulates the deimination of a small subset of the proteins making up the entire brain proteome. Additionally, it was also observed that the deimination status of certain protein features was reduced following blast exposure. Together, these observations indicate that (i) protein deimination normally takes place in the brain, (ii) aberrant deimination occurs in response to brain injury, and (iii) protein deimination may be reversible, analogous to phosphorylation. While the extent to which aberrant protein deimination contributes to injury-induced neuropathology has yet to be determined, recent findings in humans show that a history of concussions or documented brain injury is associated with the expression of brain-specific autoantibodies against GFAP [39] and S100b [43]. An important question facing this research area concerns the potential role of deimination in establishing the autoimmune response to these and other proteins following brain injury.

**Figure 5:**
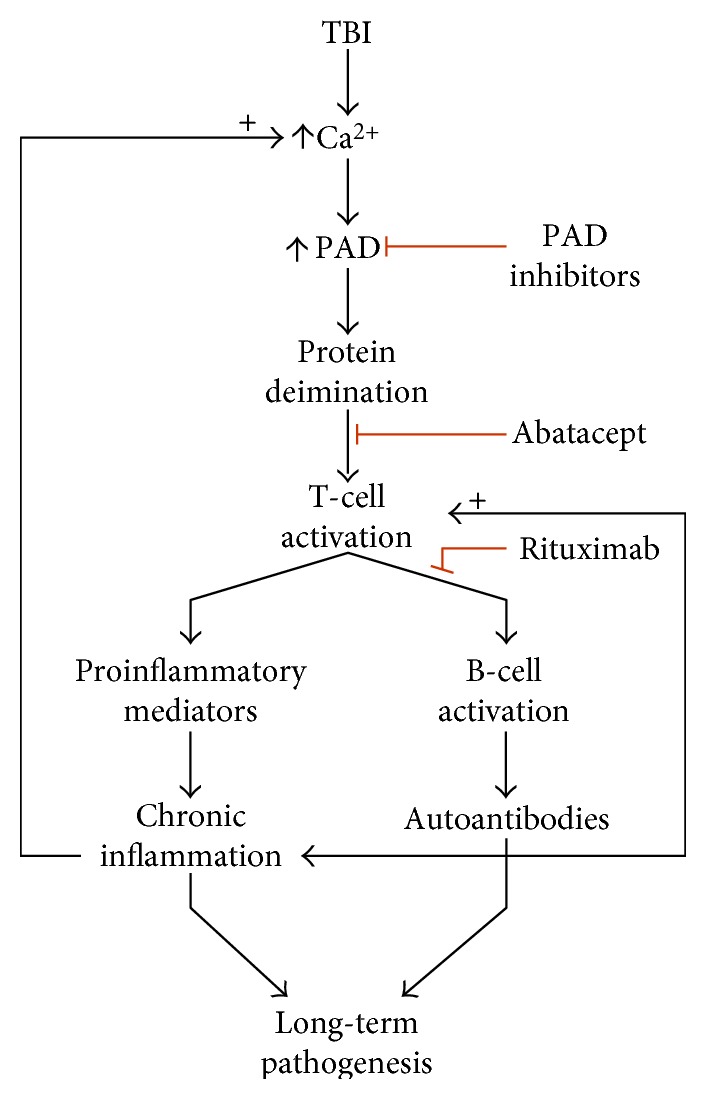
Proposed mechanism for the role of aberrant protein deimination in an autoimmune response to brain injury. TBI-induced calcium excitotoxicity hyperactivates PAD resulting in an abnormal pattern of protein deimination. Cells of the adaptive immune system process the modified proteins to reveal antigenic epitopes created by deimination. Antigen presentation and T-cell activation subsequently lead to the activation of B-cells for the production of autoantibodies and chronic neuroinflammation. It is proposed that these mechanisms contribute to long-term pathologies that can result from TBI. Potential therapeutic interventions that inhibit protein deimination and T-cell and B-cell activation are depicted with red lines.

A working model for the sequence of events that could result in a brain-specific autoimmune response to injury is presented in Figure 5. Central to this model are injury-induced oxidative stress and calcium excitotoxicity, activation of PADs and aberrant protein deimination, T- and B-cell activation in response to novel deiminated autoantigens, and the resulting establishment of a chronic inflammatory state via sustained activation of the adaptive immune system. This potential mechanism involving the adaptive immune system presents a substantial concern for long-term pathogenesis, as well as a target for therapy. Also depicted in the model are three avenues for therapeutic intervention that address the inhibition of PAD, as well as T- and B-cell activation. To date, PAD inhibitors have not been tested in humans, although one or more prototype drugs are expected to reach clinical trials soon. Recent evidence indicates that the therapeutic effectiveness of the autoimmune therapies, abatacept [44] and rituximab [45, 46], in rheumatoid arthritis is directly related to the titer of autoantibodies reactive to deiminated proteins. Accordingly, models of long-term brain injury involving an autoimmune response to deiminated proteins may benefit from these therapies.

The relations between protein deimination and the adaptive immune system in rheumatoid arthritis and neurodegenerative diseases suggest that these states may share a common mechanism. As reported for rheumatoid arthritis, multiple sclerosis [47], Alzheimer's disease [48], and prion disease [49] have distinctive profiles of protein deimination. The vimentin sequence of TVET**r**DGQVINETSQHHDDLE identified here in blast injury precisely matches the site (indicated by “**r**”) found to be deiminated in rheumatoid arthritis [40]. Moreover, the deimination site in GFAP observed here, TVEM**r**DGEVIK, was reported as a possible deimination site in Alzheimer's disease [33, 34] and the core sequence of this peptide, EM**r**DGEVIK, has also been shown to be reactive with circulating autoantibodies in a patient with relapsing-remittent multiple sclerosis [32]. On the basis of these findings, it is proposed that aberrant protein deimination and subsequent involvement of the adaptive immune system may be an underlying mechanism shared by chronic neurodegenerative diseases and classical autoimmune diseases.

Finally, this study has several limitations that should be noted. Among these are a relatively small N size (4 animals per group), the presence of only one time point (2 weeks postblast), one blast condition (40–52 psi), and one gender (male). Additionally, by necessity, the discovery proteomic analyses were performed on treatment group pools and evaluated one, albeit major brain region, the frontal cortex. As such, this research does not account for individual differences and the likelihood that blast injury effects may vary across animals and brain regions. This potential for animal variation is suggested by differences in the presence of IgGs, possibly autoantibodies, in the brain samples from individual animals (Figure 4). Nevertheless, our preliminary study identified a short list of specific proteins and their respective epitopes that can be used to focus further investigations into the questions raised here.

In summary, the research presented here shows that blast injury affects the deimination status of select brain proteins. This finding provides the basis for a mechanistic link between the acute processes of brain injury and the expression of sustained neuropathology involving activation of the adaptive immune system. The potential role for abnormally deiminated proteins in this mechanism is supported by the firmly established role for protein deimination in the hallmark autoimmune disease, rheumatoid arthritis. Recent evidence that abnormal protein deimination may play a similar role in multiple sclerosis and other neurodegenerative diseases [25, 27, 28, 50] indicates that the use of acute therapies targeting protein deimination may be of value in mitigating the long-term consequences of blast and perhaps other forms of brain injury.

## 5. Conclusions

Blast-induced brain injury can result in long-term sequelae for which there is no known underlying mechanism. Here, we propose a role for the adaptive immune system in mediating chronic pathologies of brain injury. Blast injury establishes a cellular environment that promotes aberrant protein deimination via activation of the calcium-dependent enzymes involved. The deimination modification can generate antigenic epitopes for activation of a sustained autoimmune response. The present findings show that blast exposure selectively increases the deimination of a small segment of the brain proteome, which includes proteins known to be involved in the autoimmune-based pathologies of multiple sclerosis and possibly, Alzheimer's disease. The data further demonstrate that blast injury is associated with an increase in IgG levels in the brain, possibly representing autoantibodies directed against novel deiminated protein epitopes. Together, these findings provide support for a mechanistic link between injury-induced protein deimination and pathogenic responses of the adaptive immune system.

## Figures and Tables

**Table 1:** Mapping deimination sites in brain proteins of swine exposed to repeated mild blast exposure.

Protein	Peptide sequence	Observed mass	Charge state	Expected mass with neutral loss	Mass of peak detected
GABA transaminase	LVQQPQNVSTFIN**R**PALGILPPENFVEK	1050.57	3	1036.24	1033.28
Aconitate hydratase	LN**R**PLTLSEK	391.23	3	376.89	376.67
Glial fibrillary acidic protein	ITIPVQTFSNLQI**R**ETSLDTK	802.43	3	788.10	789.75
	TVEM**R**DGEVIK	647.32	2	625.82	625.82
Glutathione S-transferase	AFLASPEHVN**R**PINGNGK	481.25	4	470.50	469.23
Histone H4	ISGLIYEET**R**GVLKVFLENVIRDAVTYTEHAK	733.80	5	725.20	726.32
Vimentin	TVET**R**DGQVINETSQHHDDLE	808.70	3	794.37	794.37

**R** = deimination site.
